# Stem-Cell-Driven Chondrogenesis: Perspectives on Amnion-Derived Cells

**DOI:** 10.3390/cells13090744

**Published:** 2024-04-24

**Authors:** Ludovica Sulcanese, Giuseppe Prencipe, Angelo Canciello, Adrián Cerveró-Varona, Monia Perugini, Annunziata Mauro, Valentina Russo, Barbara Barboni

**Affiliations:** 1 Unit of Basic and Applied Sciences, Department of Biosciences and Agri-Food and Environmental Technologies, University of Teramo, 64100 Teramo, Italy; gprencipe@unite.it (G.P.); acanciello@unite.it (A.C.); acerverovarona@unite.it (A.C.-V.); amauro@unite.it (A.M.); vrusso@unite.it (V.R.); bbarboni@unite.it (B.B.); 2 Department of Bioscience and Technology for Food, Agriculture, and Environment, University of Teramo, 64100 Teramo, Italy; mperugini@unite.it

**Keywords:** chondrogenesis, stem cells, amnion-derived cells, tissue regeneration, stem cells differentiation, cartilage regeneration

## Abstract

Regenerative medicine harnesses stem cells’ capacity to restore damaged tissues and organs. In vitro methods employing specific bioactive molecules, such as growth factors, bio-inductive scaffolds, 3D cultures, co-cultures, and mechanical stimuli, steer stem cells toward the desired differentiation pathways, mimicking their natural development. Chondrogenesis presents a challenge for regenerative medicine. This intricate process involves precise modulation of chondro-related transcription factors and pathways, critical for generating cartilage. Cartilage damage disrupts this process, impeding proper tissue healing due to its unique mechanical and anatomical characteristics. Consequently, the resultant tissue often forms fibrocartilage, which lacks adequate mechanical properties, posing a significant hurdle for effective regeneration. This review comprehensively explores studies showcasing the potential of amniotic mesenchymal stem cells (AMSCs) and amniotic epithelial cells (AECs) in chondrogenic differentiation. These cells exhibit innate characteristics that position them as promising candidates for regenerative medicine. Their capacity to differentiate toward chondrocytes offers a pathway for developing effective regenerative protocols. Understanding and leveraging the innate properties of AMSCs and AECs hold promise in addressing the challenges associated with cartilage repair, potentially offering superior outcomes in tissue regeneration.

## 1. Introduction

Cartilaginous tissue can be found in several parts of the body, playing both a structural and morphological function. The most diffused type of cartilage in the body is hyaline cartilage [1]. Articular cartilage is a special type of hyaline cartilage located in the diarthrodial joints: its role is to provide a smooth and highly lubricated surface to resist mechanical stresses and plastic deformation during movements [2,3,4]. This tissue is formed by a single cell type—the chondrocytes, which represent just 1 to 5% of the cartilage volume. Chondrocytes are a heterogeneous population that varies in number and shape based on the articular zone they reside in. These cells produce and are embedded in a dense extracellular matrix (ECM), which, in turn, provides the cells with all the necessary nutrients via diffusion from the synovial fluid [2,3,4,5,6,7,8]. Mature cartilage is composed of about 90% of ECM, which is comprised of 80% of water and 20% of a solid phase (mainly composed of collagen, proteoglycans, and glycoproteins) [1,8,9]. About 30% of water is present in the intrafibrillar space of the collagen fibres. Moreover, the small diameter of the ECM pores allows control and balance of the fluid flow, providing cartilage with a high resistance to friction and, consequently, to compressive strength [3]. Collagen represents the most abundant protein in cartilage ECM. The prevalent type of collagen in cartilage is collagen II, accounting for at least 90% of collagen molecules of this tissue. Other collagen molecules, reported in cartilage are type I, III, VI, IX, X, and XI [3,10]. Among the non-collagenous proteins that compose the cartilage ECM, proteoglycans are the most pivotal. The glycosaminoglycans (GAGs), usually present in cartilage, are hyaluronan, chondroitin 4- or 6-sulphate, keratan sulphate, and dermatan sulphate [11].

Due to these anatomical features and the lack of nerves, blood, and lymphatic vessels, cartilage is dependent on the neighbouring tissues for nutrients and oxygen supply. These anatomical features are one of the main reasons for cartilage healing failure [12,13]. Chondrocytes obtain energy mainly from glycolysis and rely on special mechanisms to adapt cell survival and differentiation to the surrounding low-oxygen (O_2_) and low-nutrient microenvironment. Once damage occurs, this tight metabolic regulation is disrupted, increasing the energy requirement that will lead to lactate accumulation and, consequently, to tissue acidification, thus easing matrix production impairment and cartilage degeneration. When the metabolic defects become chronic, the damage is irretrievable and cartilage will inevitably degenerate [13]. Another crucial feature that may explain the struggle in regenerating cartilage is the low cell density. The greatest proportion of cartilage is composed of ECM, with few chondrocytes that possess a low proliferative ability embedded in it [14]. Also, modifications of the mechanical properties influence the regeneration outcome. Indeed, a change in the loading area induces the rupture of the ECM, disrupting the collagen network and stimulating the onset of inflammation. The inflammatory environment then enhances ECM damages by stimulating the production of enzymes like metalloproteases that catabolize the ECM and reduce the survival capacity of chondrocytes [13]. Therefore, cartilage is often spontaneously repaired into a tissue that only partially resembles the healthy status, known as fibrocartilage. Fibrocartilage possesses inferior mechanical properties compared to hyaline cartilage and progressively degrades over time, representing the main issue to be overcome for the development of an effective regenerative protocol [12].

Nowadays, arthritis, characterized by the presence of joint pain and stiffness, represents the most common cause of musculoskeletal disorders (https://www.cdc.gov/arthritis/types/index.html, (accessed on 22 April 2024)). It retains a huge economic, personal, and social burden. Apart from the medical costs that are over hundreds of billions of dollars, the economic burden of this condition is connected to indirect costs, such as the loss of productivity. The term “arthritis” gathers a group of more than 100 inflammatory conditions affecting the joint and the neighbouring tissues [15]. The most diffused forms of arthritis are rheumatoid arthritis and osteoarthritis, both characterized by the presence of inflammation, remodelling of the bone structure, and degeneration of the articular cartilage [16]. The actual management guidelines for cartilage repair suggest the combination of non-pharmacological and pharmacological interventions. When both types of interventions fail, the only remaining solution is surgery to totally replace the joint in end-stage pathologies or to restore the normal joint and minimize the damage in early-stage symptomatic cartilage damages. Surgical interventions include three main techniques: microfracture, autologous chondrocyte implantation (ACI), and osteochondral auto- or allografts [17]. Recently, new strategies for cartilage engineering have been proposed. These methods have the purpose of generating mature functional cartilaginous tissue by exploiting the guide of a scaffold that resembles the biomechanical features of cartilage in in vitro cultures. The most innovative strategy in this field is the one combining the use of scaffolds and the ACI techniques known as matrix-associated autologous chondrocyte implantation (MACI) [18,19,20]. Nevertheless, the current strategies for osteoarthritis management are not sufficient and do not block the disease incidence and progression. More efforts should be made to develop new cutting-edge therapies and to promote disease prevention activities [21,22,23].

A growing interest has been directed towards regenerative medicine, with a particular focus on stem-cell-based therapies. Regenerative medicine exploits the potential of stem cells to functionally restore damaged tissues and/or organs in patients suffering from severe untreatable diseases [24,25]. Cell therapies involve the engraftment of autologous stem cells (stem cells from the same patient), allogenic stem cells (stem cells from a healthy donor), or syngeneic stem cells (stem cells from a genetically identical donor). Once engrafted, these cells should not only survive, proliferate, and differentiate but should also be able to integrate into the recipient tissue [24]. The only cell source currently utilized and approved by the Food and Drug Administration (FDA) and the European Medicines Agency (EMA) for the clinics are the chondrocytes, but various methods based on different types of stem cells have been experimented and are under the agencies’ scrutiny [18,19,20]. Interestingly, the EMA, in 2017, approved the use in the clinics of a spheroid-based therapy named “spheroids of human autologous matrix-associated chondrocytes” (Spherox), which allows the treatment of large articular cartilage defects of the knee (https://www.ema.europa.eu/en/medicines/human/EPAR/spherox, (accessed on 22 April 2024)). Recently, stem cell research is focussed towards the use of perinatal stem cells, a promising source for regenerative medicine applications. These cells are derived from extra-embryonal tissues, such as foetal membranous structures (amnion, allantois, chorion, and yolk sac) and the umbilical cord (Wharton’s jelly, umbilical cord membrane, veins, and arteries) [26]. Perinatal stem cells are easy to harvest and are free of ethical or biological constraints usually associated to other sources of stem cells. They are characterized by high plasticity, in particular, those derived from early embryonic-derived structure, such as amnion [27]. Amnion-derived stem cells, such as amniotic epithelial cells (AECs) and amniotic mesenchymal stem cells (AMSCs), express embryonic markers and possess pluripotent characteristics. Indeed, they combine high self-renewal with great plasticity [28,29]. Moreover, large scientific evidence demonstrated that these cells are clinically safe, are not teratogenic, and can be used for allo- and xeno-transplantation in immunocompetent patients due to their “immune privilege” [27].

The aim of this review is to discuss the potential of AMSCs and AECs to differentiate towards the chondrogenic lineage, with a focus on the pathways involved in chondrogenic differentiation.

## 2. Scientometric Analysis

**Figure 1:**
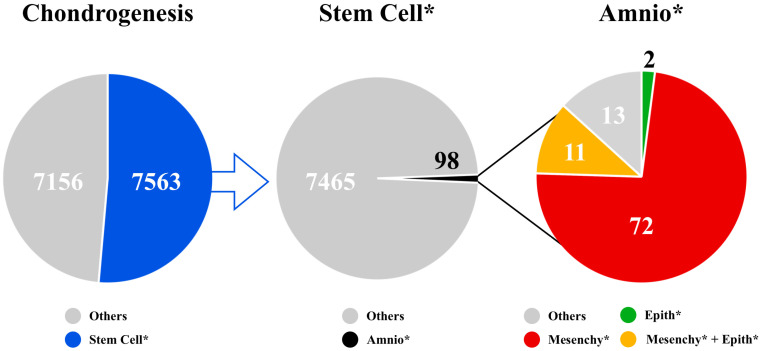
Comparative scientometric analysis of the available publications on the Scopus database found using the keywords “chondrogenesis”, “stem cell*”, “amnio*”, “epith*”, and “mesenchy*”: 7563 (51.3%) of the total publications on chondrogenesis concern stem cells. A deeper analysis of stem cells origin revealed that only 98 (1.3%) of the publications concern amnion-derived stem cells. Furthermore, 72 (73.5%) of the latter regard amniotic mesenchymal stem cells, 2 (2%) amniotic epithelial cells, and 11 (11.2%) concern both cell types.

The relevance of the topic can be inferred by performing a Scopus database analysis with a scientometric approach on the available literature, using “chondrogenesis” as a keyword. The analysis revealed that 14,719 papers have been published on this topic. Then, when focusing on researching the keyword “stem cell*”, the outcome of the database analysis revealed that only about half (7563, 51.3%) of the articles concerning chondrogenesis involve the topic of stem cells. Specifically, we went further to determine how many articles refer to amnion-derived stem cells by using the keywords “amnio*”, “epith*”, and “mesenchy*”. The research revealed that just 98 (1.3%) articles refer to “amnio*”. From those, two (2%) refer to “epith*” alone, seventy-two (73.5%) to “mesenchy*”, and eleven (11.2%) to both, for a total of eighty-five articles, indicating the novelty of the topic (Figure 1). Among them, 32 articles have been selected, all concerning the techniques utilized to induce chondrogenic differentiation in AECs and AMSCs. Articles that were concerned with embryonic stem cells, induced pluripotent stem cells, or amniotic fluid stem cells and that were not related to articular cartilage were excluded. Starting from these premises, this review explores the molecular pathways that coordinate chondrogenesis in stem cells and the various methods tested to reach chondrogenic differentiation in both AMSCs and AECs.

## 3. Pathways Regulating Chondrogenesis

**Figure 2:**
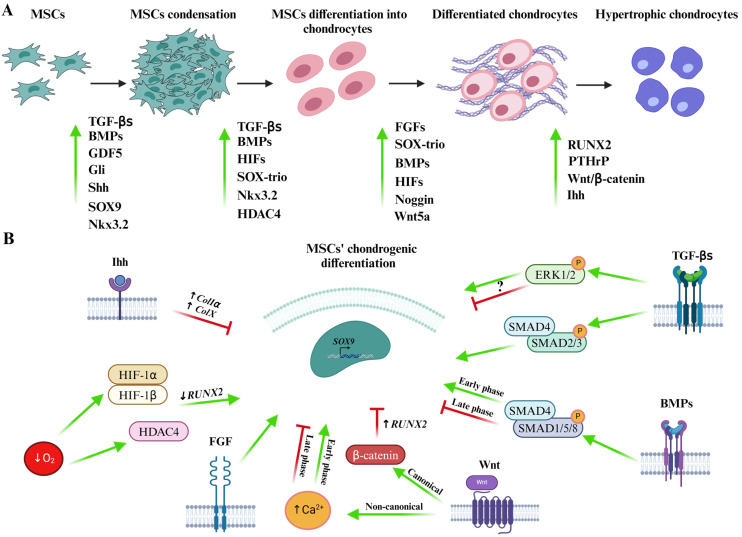
Summary of pathways involved in chondrogenesis. (**A**) Molecules involved in the various phases of MSCs chondrogenesis. (**B**) Promoting and inhibitory factors that collaborate during MSCs chondrogenic differentiation via *SOX9* expression. *SRY-Box Transcription Factor (SOX)*; *collagen (Col)*; *Runt-related Transcription Factor 2 (RUNX2)*; Growth Differentiation Factor 5 (GDF5); Wingless-related integration site (Wnt); Small Mothers Against Decapentaplegic (SMAD); Transforming Growth Factor-beta (TGF-β); Bone Morphogenetic Protein (BMP); Indian Hedgehog (Ihh); Fibroblast Growth Factor (Fgf); Hypoxia Inducible Factor 1 Alpha (HIF1α); Hypoxia Inducible Factor 1 Beta (HIF1β); Histone Deacetylase 4 (HDAC4); oxygen (O_2_); Extracellular Signal-Regulated Kinase (ERK); Sonic Hedgehog (Shh); *NK3 Homeobox 2 (Nkx3.2)*; mesenchymal stromal cells (MSCs); Glioma-Associated Oncogenes (Gli); Noggin; Parathyroid Hormone-related Protein (PTHrP).

Chondrogenesis is a stepwise cell differentiation process: the early phase is characterized by cell condensation and expression of the transcription factor “*SRY-related high mobility group-box gene*” *(SOX) 9*; subsequently, developing chondrocytes, that already release collagen II, start to progress towards the mature chondrocyte state, characterized by the production of an abundant ECM composed of GAGs, aggrecan, and collagen II [30,31]. *SOX9* works in synergy with two other members of the SOX family, namely *SOX5* and *SOX6*, constituting the *SOX*-trio genes. Evidence suggested that only the *SOX*-trio can successfully induce chondrogenic differentiation in all cytotypes. SOX9 allows the expression of downstream genes regulating the deposition of components of chondro-related ECM such as *collagen II*, *IX*, and *XI*; *aggrecan*; *cartilage link protein (CRTL1)*; and *cartilage oligomeric matrix protein (COMP)* by binding to the promoter region of their genes or forming trans-activating complexes [31,32]. *SOX9* expression is regulated by a series of upstream pathways that are implicated in directing stem cell differentiation into chondrocytes: Transforming Growth Factor-beta (TGF-β)/Bone Morphogenetic Protein (BMP), Wingless-related integration site (Wnt)/β-catenin, and Mitogen-Activated Protein Kinase (MAPK) [33]. Several studies have been performed in vivo by developing knock out (KO) mice models to understand the involvement of these different pathways in regulating chondrogenesis. A summary is reported in Figure 2 and Table 1.

### 3.1. Chondrogenesis during Embryogenesis

**Figure 3:**
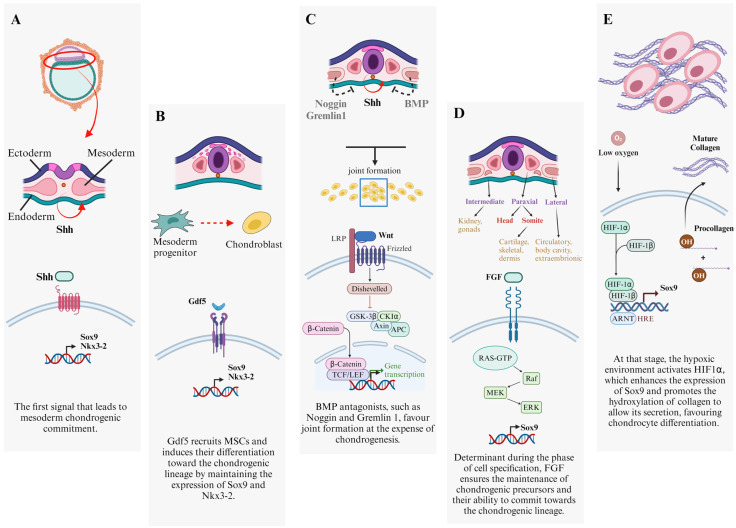
Summary of cartilage development. (**A**) Activation of chondrogenic signals thanks to mesoderm specification from Shh. (**B**) Beginning of cell condensation, stimulated by Gdf5 production. (**C**) Development of joints and activation of BMP antagonists. (**D**) Upregulation of FGF signalling to maintain cell specification along chondrogenesis. (**E**) Upregulation of HIF1α to further enhance the expression of chondro-related genes (*Sox9*, *aggrecan*, and *collagen*). Sonic Hedgehog (Shh); *SRY-Box Transcription Factor 9 (Sox9)*; *NK3 Homeobox 2 (Nkx3.2)*; Growth Differentiation Factor 5 (Gdf5); mesenchymal stromal cells (MSCs); Bone Morphogenetic Protein (BMP); Glycogen Synthase Kinase 3 Beta (GSK-3β); Casein Kinase 1 Alpha (CK1α); Adenomatous Polyposis Coli (APC); T-cell Factor (TCF)/Lymphoid Enhancer Factor (LEF); Fibroblast Growth Factor (FGF); Rat Sarcoma (RAS)-Guanosine Triphosphate (GTP); Rapid Accelerated Fibrosarcoma (Raf); Mitogen-Activated Protein Kinase (MEK); Extracellular Signal-Regulated Kinase (ERK); oxygen (O_2_); Hypoxia Inducible factor (HIF); Aryl Hydrocarbon Receptor Nuclear Translocator (ARNT); *Hypoxia Response Element* (HRE); hydroxyl group (OH).

Cartilage derives from the embryonic germ layer mesoderm, which is responsible for the generation of the appendicular skeleton from the fourth week of gestation. Chondrogenesis is carried out by mesenchymal stromal cells (MSCs) and is accompanied by their condensation via cell–cell interaction and, later, cell–matrix interaction. These interactions lead to the generation of a high-density cell mass (Figure 3A). The latter, in turn, gives rise to the cartilage anlagen, characterized by the presence of prechondrogenic cells. The condensation process is characterized by the activation of hyaluronidase and of cell adhesion molecules such as N-cadherin and the “neural cell adhesion molecule” (N-CAM). Importantly, the grade of condensation will determine the rate of chondrogenic differentiation [58,59]. The first signal that leads to mesoderm chondrogenic commitment is provided by the Sonic Hedgehog (Shh) pathway. Shh signalling activates the Glioma-Associated Oncogenes (Gli) transcription factors (Gli1, Gli2, and Gli3) that, in turn, trigger the expression of the hedgehog-responsive genes, which stimulates the initial expression of SOX9 and “NK3 Homeobox 2” (NKX3.2). The latter is a transcription factor that indirectly maintains SOX9 expression, by blocking the chondro-inhibiting transcription factors “GATA Binding Protein” (GATA) 4, 5, and 6 [60,61]. Nevertheless, the Shh signal is necessary only for the primordial induction of mesodermal differentiation (Figure 3A). Indeed, condensation is initially stimulated by BMP signalling, in particular by “Growth and differentiation factor 5” (GDF5), which recruits MSCs and induces their differentiation toward the chondrogenic lineage by maintaining the expression of SOX9 and NKX3.2 (Figure 3B) [61,62]. The intervention of BMP antagonists such as Noggin is also crucial, which favours joint formation at the expense of chondrogenesis. Another pivotal BMP-antagonist is gremlin 1 which adopts the Noggin task after birth (Figure 3C) [61,62,63]. Likewise, “fibroblast growth factor” (FGF) signalling acts as a determinant during this phase of cell specification by ensuring the maintenance of chondrogenic precursor cells’ viability and of their competence to commit towards the chondrogenic lineage. Particularly, FGF signalling enhances the expression of SOX9 in chondrocyte precursors through the activation of the MAPK pathway (Figure 3D) [61]. Another factor involved in the initial stages of chondrogenesis is HIF1α. HIF1α enhances the expression of SOX9 and promotes the hydroxylation of collagen to allow its secretion, favouring chondrocyte differentiation (Figure 3E). Cells in the cartilage anlagen may differentiate into two cytotypes: the persistent chondrocytes (persistent cartilage) and the proliferating chondrocytes (transient cartilage). The former synthesize a wide amount of ECM and collagen to ultimately form the hyaline cartilage; the latter undergo hypertrophy and give rise to the growth plate. At first, hypertrophic chondrocytes stimulate the formation of the primary ossification centre. Hypertrophic chondrocytes enlarge, switch collagen expression from II to X, mineralize the surrounding matrix, produce pro-angiogenic factors and matrix metalloproteases (MMPs), and undergo apoptosis. This process triggers vascular invasion, allowing bone precursor cells to remodel the matrix into bone tissue. Then, the growth plates (composed of the remaining cartilage segments at both sides of the primary ossification centre) drive longitudinal bone growth [59]. After the generation of these structures, a mesenchymal interzone develops where cartilage will remain enclosed and future synovial joints will form, dividing adjacent skeletal components. Postnatally, the secondary ossification centre arises in the epiphyseal regions, establishing the boundary between the growth plate and the region of articular cartilage, allowing the lengthening of the bone [59,64]. The cartilage near the secondary ossification centre and the joint cavity will persist as articular cartilage [63]. Cells that commit towards the chondrogenic lineage gain a spherical shape (cartilage primordia) and start to express the transcription factors SOX9, SOX5, and SOX6. [59,61]. Then, chondrocytes that undergo hypertrophy, will be directed by “Indian Hedgehog” (Ihh) and “parathyroid hormone-related protein” (PTHrP) pathways to express the early hypertrophic markers collagen Iα and X. The expression of these markers is correlated with the loss of SOX9, SOX5, and SOX6; collagen IIα1; and aggrecan expression, leading to differentiation into mature hypertrophic chondrocytes [61].

### 3.2. Morphogenic Pathways: TGF-β/BMP and Shh Pathways

The TGF-β molecular pathway is composed of two subfamilies of factors: the TGF-β and the BMP family members. The TGF-β/BMP signalling pathways exert a pivotal role in chondrogenic differentiation, but their effects depend on the stage of chondrocyte differentiation (Figure 2A) [60,65,66]. Indeed, TGF-β family members, at the early stages of chondrogenic differentiation, initiate a series of signalling cascades that will induce SOX9 gene expression (Figure 2B) [67,68,69,70]. The signalling cascade begins with TGF-β ligand expression that triggers signalling cascades at the cell membrane by attaching and organizing a receptor complex on the surface of the cell. This complex is formed by the interaction of type II and type I serine/threonine kinase receptors. Usually, TGF-β binds to the type II receptor TGFβR2, which subsequently phosphorylates the type I receptor TGFβR1/Activin receptor-like kinase (i.e., ALK5). In the canonical pathway, TGFβR1 phosphorylation induces, in turn, Small Mothers Against Decapentaplegic (SMAD) 2/3 complex (TGF-β specific Smads) activation through phosphorylation (Figure 2B) [65,66,67,69,71,72,73]. SMAD2/3 activity is crucial for the entire process of chondrogenesis, and it has been demonstrated that it blocks chondrocytes terminal differentiation, promoting collagen II deposition for the production of stable hyaline cartilage. Interestingly, SMAD2 and SMAD3 exert a different function to mediate TGF-β signalling. Indeed, it seems that SMAD3, by directly binding to DNA, has a prevailing impact on SMAD2, which controls gene expression by interacting with SMAD3 or alternative transcription factors [65,69,71,72,73]. However, the activation of TGFβR1 may also lead to the activation of non-canonical, SMAD-independent pathways. For instance, it was found that, through the non-canonical pathway, TGF-β can activate the MAPK pathways, including the activation of Extracellular Signal-Regulated Kinase (ERK). Interestingly, the MAPK and SMAD signals may cooperate for the activation of the same targets. Concerning the relationship between ERK and SMAD for chondrogenic induction, Luo et al. demonstrated that during human AMSCs (hAMSCs) chondrogenic differentiation both SMAD2/3 and ERK signalling were upregulated during early chondrogenesis. Nonetheless, the role of ERK in chondrogenesis is still controversial, since it seems to have both promoting and inhibitory effects (Figure 2B) [65,72]. Upon the activation of different types of type I receptors (i.e., ALK1), the activation of the BMP signalling pathway occurs. BMP signalling is pivotal not only during the early phases of differentiation, but also for the maintenance of the cytotype later on. Once activated, the receptors induce phosphorylation of the SMAD1/5/8 complex that will bind to SMAD4, eventually leading to SOX9 expression (Figure 2B). The SMAD 1/5/8 pathway is necessary for chondrocyte terminal differentiation. Importantly, the role of SMAD 2/3 and SMAD 1/5/8 is stage dependent. The phosphorylation of both is necessary for the early stages of chondrogenesis, while in later stages their role becomes divergent [60,71,73]. The relationship between TGF-βs and BMPs in chondrogenesis is necessary to efficiently complete the process. It was found that TGF-β amplifies BMP2-triggered chondrogenesis, enhances the hyaline-like characteristics of newly formed cartilage, and halts differentiation at the initial phases of hypertrophy. On the contrary, BMP2 notably diminishes TGF-β signalling levels. These findings imply that during early chondrogenesis TGF-β fosters BMP signalling [74,75]. BMP4 activity follows the activation of the Shh pathway to enhance the expression of chondrogenesis-related genes. Importantly, Shh and BMP cooperation has to be sequential and not simultaneous, since in the last case BMP would block chondrogenesis by inducing the expression of the GATA transcription factors [60,61].

### 3.3. Morphogenic Pathways: Wnt/β-Catenin Pathways

The Wnt/β-catenin signalling pathways comprise both the canonical and non-canonical Wnt. The canonical pathway is the one implicated in chondrocytes’ hypertrophy induction. It exerts its functions by controlling β-catenin fate through the receptor Frizzled. When canonical Wnt is absent, β-catenin is degraded by phosphorylation-mediated ubiquitination after binding to a degradation complex made up of “glycogen synthase kinase 3” (GSK3), “adenomatous polyposis coli” (APC), Axin, and “casein kinase 1α” (CKI). Once canonical Wnt is activated, it binds to the receptor Frizzled and blocks the activity of the degradation complex, freeing β-catenin. This event allows β-catenin to enter the nucleus, where it binds to “lymphoid enhancer factor” (LEF) and “T-cell factor” (TCF) transcription factors. This complex favours RUNX2 expression leading to hypertrophy (Figure 2B). When SOX9 is expressed, it will induce β-catenin degradation via phosphorylation and ubiquitination, preventing RUNX2 expression [67]. Interestingly, canonical Wnt signal represses SOX9 expression by acting at the epigenetic level: it causes methylation at the chromatin level on the lysin 27 of histone 3 (H3K27me3) and on the DNA at the SOX9 promoter (Figure 2B) [61]. Non-canonical Wnts, as Wnt5a, possess two different functions in stem cells chondrogenic differentiation. During the early phase, they stimulate intracellular calcium (Ca^2+^) release, promoting chondrogenesis (Figure 2B). They inhibit hypertrophy by activating the Phosphoinositide 3-Kinase (PI3K)/Protein Kinase B (Akt) pathway that will induce the expression of “Nuclear factor kappa-light-chain-enhancer of activated B cells” (NF-kB), a potent inhibitor of RUNX2. Conversely, Wnt5a later promotes chondrogenesis and inhibits SOX9 expression. These data suggest Wnt5a involvement in stage-dependent regulation of chondrogenesis and hypertrophy through distinct signalling pathways [67,69,76]. Interestingly, a crosstalk exists between TGF-β/BMP and canonical Wnt signalling, creating a feedback loop to mutually regulate their activities. The TGF-β and Wnt signalling pathways act either independently or collaboratively to regulate the expression of LEF1/TCF target genes. Upon TGF-β stimulation, SMAD3 forms interactions with LEF1 to initiate the transcription of target genes. Furthermore, TGF-β additionally enhances the nuclear accumulation and stability of β-catenin. Therefore, collaborating with Wnt signalling pathways, TGF-β promotes the differentiation of chondrocytes from mesenchymal cells [67,77,78].

### 3.4. Other Mechanisms

#### 3.4.1. Low O_2_ Tension

Low O_2_ tension seems to possess a critical role in chondrogenesis. In particular, it has been proved to stimulate chondrogenesis in stem cells and restore chondrogenic phenotype in de-differentiated chondrocytes. Indeed, low O_2_ tension induces the expression of the transcription complex HIF that contains HIF1α and HIF2α subunits. The complex binds to the ”hypoxia-response element” (HRE), an 8 bp hypoxia-responsive element, which promotes chondrogenesis by stimulating the expression of SOX9 and aggrecan (Figure 2B). HIF1α responds to oxygen levels and enhances the expression of glycolytic enzymes and glucose transporters, favouring the differentiation and adaptation of chondrocytes to a low O_2_ environment. The β-subunit of HIF1, also called the “aryl hydrocarbon receptor nuclear translocator” (ARNT), is continuously expressed. It downregulates the expression of hypertrophic and osteogenic markers by blocking the binding activity of the “core binding factor-a1” (CBFA1)/RUNX2 to the collagen X promoter [61,67,70,79]. A pivotal function of HIF1 is to protect cells surrounded by a low O_2_ environment from apoptosis [79]. Furthermore, low O_2_ tension enhances SOX9 expression and increases collagen II deposition in articular cartilage chondrocytes cultured in vitro. Interestingly, HIF1α generates a crosstalk between BMP2, TGFβ, and “Insulin-like growth factors” (IGFs), suggesting that HIF1 may possess a crucial role in maintaining the phenotype of chondrocytes [79,80,81]. Moreover, overexpression of HIF1α after transduction resulted in improved outcomes for cartilage regeneration [82].

#### 3.4.2. Epigenetic Regulation

Epigenetic regulation through histone acetylation/deacetylation and miRNAs plays a critical role in chondrogenic differentiation. For instance, the increased activity of the Histone Deacetylase (HDAC) 4 strongly increases chondrogenesis and parallelly blocks the onset of hypertrophy by inhibiting RUNX2 and “myocyte enhancer factor 2C” (MEF2C) expression. HDAC5 and HDAC7 suppress the expression of the above-mentioned transcription factors, favouring chondrogenic differentiation [67]. Among miRNAs, miR-140 should be mentioned, whose expression acts parallel to SOX9 expression; it is able to chondrogenically induce MSCs. Nakamura et al. found out that miR-140 is abundantly expressed in chondrocytes and proved that it is a pivotal regulator of cartilage homeostasis [57,67]. Several targets of miR-140 have been reported. They are mainly involved in the regulation of matrix degradation, chondrocytes hypertrophy, and senescence. Interestingly, miR-140 inhibits cartilage hypertrophy by targeting HDAC4 and SMAD1 [83,84,85]. miR-140 is downstream of SOX-trio activation and simultaneously it favours chondrogenesis. On the contrary, the TGF-β/SMAD3 pathway inhibits miR-140 expression, favouring matrix catabolism [86,87]. Interestingly, miR-675 is highly specific and synthesized in cartilage. miR-675 is expressed downstream of the SOX9 pathway, and it is implicated in the regulation of collagen IIa1 upregulation [88]. miR-145 is also noteworthy, which acts as a direct regulator of SOX9. Its upregulation inhibits SOX9 expression in chondrocytes along with collagen IIa1 and aggrecan [89]. Also, miR-29 is implicated in the regulation of cartilage development and homeostasis. Its expression is inversely related to SOX9 synthesis, and it is downstream of the TGF-β1 pathway. miR-29 and TGF-β1 mutually regulate their activity via a feed-forward loop, where TGF-β1 downregulates miR-29 that, in turn, inhibits the SMAD pathway, favouring the regulation of SMAD-dependent genes. Downregulation of miR-29 via TGF-β1 may be required for the maintenance of cartilage homeostasis by promoting chondrocytes proliferation. miR-29 directly targets the collagen IIa1 gene, suppressing its expression in cartilage ECM and blocking the development of chondrocytes’ mature phenotype. It also showed a function in inhibiting the Wnt signalling pathway [90,91].

## 4. Failure of Chondrogenic Mechanisms in Hypertrophic Chondro-Healing

Interestingly, functional mature chondrocytes can be achieved by balancing pro- and anti-chondrogenic signals. Indeed, stem/progenitor cells exposed to improper stimuli may achieve a hypertrophic phenotype that is indicative of a propension to furtherly progress towards the endochondral bone tissue lineage. This event is undesirable for articular cartilage, since it reduces the number of functional chondrocytes and leads to focal calcification. The anti-chondrogenic stimuli is crucial for maintaining the functional chondrocyte phenotype, required to provide high-quality cartilage-regenerated tissue [67,70].

Cartilage hypertrophy repair is characterized by ECM mineralization and increases in cell volume followed by chondrocyte’s apoptosis. This process is due to the activation of RUNX2 and MEF2, two transcription factors involved in endochondral ossification. Their expression stimulates the production of collagen type X, MMP13, “alkaline phosphatase” (ALP), and “vascular endothelial growth factor” (VEGF), which are responsible for ECM remodelling and calcification [67,70]. Moreover, the interaction between mature chondrocytes and the ECM through the integrin signalling pathway may lead to hypertrophy. Indeed, integrin signalling leads to the activation of Rho family proteins, which are responsible for actin cytoskeleton organization. Therefore, activation of the integrin–Rho signalling pathway stimulates actin cytoskeletal remodelling into focal adhesions and stress fibres and induces the activation of the p38/MAPK pathway [67]. To achieve full differentiation into a non-hypertrophic phenotype, many strategies have been proposed. Firstly, the activation of Noggin, a BMPs antagonist, along with Wnt-4, Wnt-14, Wnt-16, and β-catenin proved to be effective to solve that issue. Another method proposed is the maintenance of SOX9 expression. Indeed, SOX9 not only may bind to β-catenin and block canonical Wnt signalling but can also directly link to RUNX2, blocking its activity and the expression of genes normally upregulated during the hypertrophic state, including collagen Xα1 and VEGF-A [61,70].

## 5. Amnion-Derived Stem Cells in Chondrogenesis

The new frontier of regenerative medicine is emerging to be the use of amnion-derived stem cells. These cells are derived from the amniotic membrane (AM), the membrane directly in contact with the foetus during pregnancy. The AM is a thin, avascular membrane that limits the amniotic cavity, composed of both epithelium and mesenchyme. Freshly isolated AMs and amnion-derived stem cells were both used for regenerative purposes [92].

AECs are derived from the AM innermost layer, directly meeting the foetus and the amniotic fluid, whilst AMSCs are dispersed throughout the whole membrane [26,93]. Those cells can easily be harvested by mechanically separating the AM from the chorion. The early embryonic origin of the epithelial layer of AM supports the hypothesis that AECs maintain a great plasticity and an embryonic phenotype [94,95,96]. Human AECs (hAECs) were, indeed, positive for the expression of classic surface markers of embryonic stem cells (ESCs), such as “T-cell receptor alpha locus” (TRA) 1-60, TRA1-81, and “stage-specific embryonic antigen” (SSEA)-4, and of pluripotent stem cells, such as “octamer-binding transcription factor” (OCT)-4, SOX2, and NANOG [94,96,97]. Also, ovine AECs (oAECs) generated embryoid bodies able to differentiate into different mesenchymal lineages, including the chondrogenic lineage [98]. Successful differentiation towards the chondrogenic lineage has been obtained from ovine, equine, and feline AECs [99,100,101].

The hAMSC phenotype is similar to that of bone marrow-MSCs (BM-MSCs), being positive for the expression of mesenchymal markers as CD73, CD90, and CD105. hAMSCs represent a good cell source for transplantation since they lack the presence of “human leukocyte antigen” (HLA)-DR and have a low expression of HLA-ABC. Moreover, experiments in vivo demonstrated their persistency and efficacy after being engrafted in animal models. hAMSCs express pluripotency and embryonic markers such as OCT4, SOX2, “Reduced expression 1” (REX1), NANOG, SSEA-3, -4, TRA 1-60, and TRA 1-81. hAMSCs are able to differentiate towards all the three germ layers, as proven by several pluripotency studies in vitro. Indeed, they express multiple lineage markers, among which chondrogenic markers can be highlighted. It has also been demonstrated that the stromal layer of the AM produces factors that control MSCs differentiation, suggesting the self-intrinsic ability of this cell source to generate mesodermal derived tissues [93,95,96]. Kim et al. reported that hAMSCs are positive for typical MSCs markers such as “alpha-smooth muscle actin” (α-SMA); vimentin; desmin; and collagen I, III, and IV and for markers typical of chondrogenesis such as collagen II and BMP4. These outcomes demonstrate the innate potential of hAMSCs to differentiate towards the chondrogenic lineage [102]. Interestingly, the possibility to transplant AM to successfully regenerate cartilage into a full-thickness femoral cartilage lesion has been reported in an ovine model [103]. Few studies proved the in vivo chondro-regenerative potential of AECs and AMSCs. It has been demonstrated that oAECs are effective in repairing cartilage defects in osteoarthritis ovine models. After three months from the injection of oAECs in the damaged cartilage, mechanical properties were improved and, parallelly, the macroscopic surface anatomy was rescued. In addition, a significant reduction in the inflammatory state of the synovial fluid was reported. In a following study, they also confirmed the results obtained 6 months after the beginning of the treatment [104,105]. the direct use of the AM to repair cartilaginous damages in vivo is also worth mentioning. Various studies demonstrated the efficacious properties of the AM in promoting cartilage reconstitution and repair after being stimulated by chondrogenic media or being placed in co-culture with cartilage particles or chondrocytes. This is true especially for the stromal/mesenchymal layer of the AM. The engraftment of the AM, after being conditioned using one of the mentioned techniques, into animal models such as rabbits or sheep with osteochondral defects led to the production, after 2–4 months, of a hyaline-cartilage-like tissue that completely filled the damage [103,106,107]. Furthermore, AECs and AMSCs possess potent paracrine effects, whose activity is responsible for the activation of tissue regenerative mechanisms thanks to the secretion of growth factors, cytokines, and exosomes. In addition, AECs and AMSCs are highly capable of transdifferentiation after chemical induction, biological treatment, or co-culture with other cell types and gene transfection. The unique properties of AECs and AMSCs suggest that they may be the perfect cell source for cartilage repair, generating a beneficial environment for tissue regeneration by supporting cell survival and activating endogenous mechanisms of tissue regeneration [26,93]. Despite their incredible technical advantage for tissue engineering and their great chondro-plasticity demonstrated in vitro, AECs and AMSCs have not yet been exploited for chondro-regenerative purposes.

### In Vitro Amnion-Derived Stem Cells Lesson on Chondro-Related Pathways

**Figure 4:**
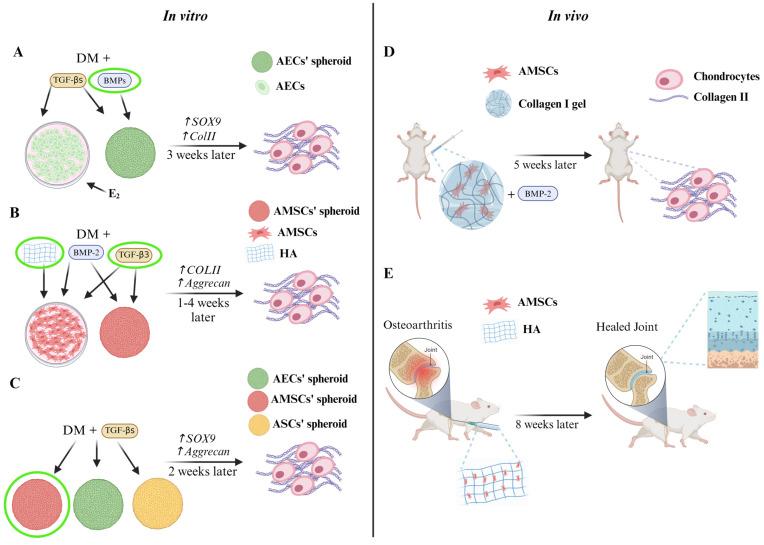
Strategies for in vitro and in vivo induction of AECs’ and AMSCs’ chondrogenic differentiation. (**A**) Chondrogenic differentiation of AECs. (**B**) Chondrogenic differentiation of AMSCs. (**C**) Comparison of AECs, AMSCs, and ASCs chondrogenic potential. (**D**) In vivo chondrogenic differentiation of AMSCs in a mouse model after engraftment within abdominal muscles. (**E**) Recovery of osteoarthritic knee after hyaluronic acid and AMSCs injection in a rat model. The icons circled in green represent the best strategies for in vitro chondrogenic induction. Amniotic epithelial cells (AECs); amniotic mesenchymal stem cells (AMSCs); adipose-derived stem cells (ASCs); chondrogenic differentiation medium (DM); Transforming Growth Factor-beta (TGF-β); Bone Morphogenetic Protein (BMP); *SRY-Box Transcription Factor 9 (SOX9)*; *collagen II* (*ColII*); hyaluronic acid (HA).

Several strategies have been developed to induce stem cell chondrogenic differentiation in vitro. The process of in vitro chondrogenic differentiation mimics the natural process of endochondral bone formation. Therefore, chondrogenic differentiation of stem cells can be achieved by treating cells with specific growth factors and by modulating the properties of the surrounding environment (i.e., low O_2_ tension, biomechanical stimulation, etc.). Moreover, the use of scaffolds and 3D cultures stimulated a great interest in the scientific community, since they better resemble in vivo joint development. At any rate, the main challenge for cartilage tissue engineering is to yield high quality, functional chondrocytes in vitro [67,70]. Traditionally, stem cells are simultaneously treated with one of the TGF-β family members (GDF5, TGF-β1, TGF-β2, TGF-β3, or BMP-2) and IGF-1 to initiate chondrogenic differentiation. The first member of the TGF-β family to be discovered was GDF5, a factor that showed both chondro-inductive and proliferative properties. Park et al. reported that TGF-β3 alone was capable of stimulating chondrogenic differentiation in MSCs [108]. Moreover, data showed that a combination of TGF-β3 with the activation of β-catenin stimulates chondrogenesis and avoids hypertrophic degeneration in MSCs. Conversely, cells treated with TGF-β1 showed signs of hypertrophy [70,108]. A study from Zhou et al. proposed to induce hAECs’ chondrogenic commitment by exposing the cells to 100 ng/mL BMP-7 for 21 days. BMP-7 is expressed in the adult articular cartilage, and its activity in vitro enhances chondrogenic differentiation and the synthesis of proteoglycans and collagen in the ECM. Zhou et al. compared the effects of cell treatment with BMP-7 or with TGF-β1 (1 ng/mL). After three weeks of induction, they observed an increased level of *SOX9* and *collagen II* expression in the BMP-7-treated group compared to the TGF-β1 group. Furthermore, BMP-7-treated hAECs differentiated into normal chondrocytes with a round shape and a collagen- and GAGs-rich ECM. Thus, Zhou et al. suggested that BMP-7 promotes the in vitro synthesis of a high-quality ECM better than the classic inducer TGF-β1 (Figure 4A) [109]. In another study, Nogami et al. compared the chondrogenic effect of TGF-β3 and BMP-2 on hAMSCs. Their data showed that only cells treated with BMP-2 were able to produce proteoglycans and the collagen IIb isoform. The latter represents the main marker of mature chondrocytes, suggesting that BMP-2 could be an efficient growth factor to differentiate hAMSCs towards the chondrogenic lineage. However, they also reported the expression of collagen I in cells treated with both TGF-β3 and BMP-2, suggesting that these factors may allow unfavourable progression toward the hypertrophic phenotype (Figure 4B) [110]. To allow stem cell differentiation in vitro, cells are cultured into a tailored differentiation medium for a precise timeframe. Generally, chondrogenic induction medium is composed of high glucose “Dulbecco’s modified Eagle medium” (DMEM), “Minimum Essential Medium Eagle-Alpha Modification” (α-MEM), or classic DMEM supplemented with insulin, selenous acid, transferrin (6.25 µg/mL each), 10^−7^ M dexamethasone, 0.1 mM ascorbic acid, and 10 ng/mL of TGF-β1, TGF-β3, or BMP-2/7. Usually, the induction lasts for 21 days, and the medium is replaced every 2–3 days. Some of the factors contained in the differentiation medium play a pivotal role in chondrogenesis. For instance, dexamethasone and insulin promote differentiation by directly stimulating the expression of transcription factors critical for MSCs commitment; ascorbic acid acts as co-factor for the hydroxylation of lysine and proline residues in collagen, promoting the maturation and deposition in the ECM of this crucial marker of chondrogenesis [68,110,111,112,113,114,115]. Wei et al. demonstrated the potency of the “recombinant human bone morphogenetic protein 2” (rHuBMP-2) to induce chondrogenic differentiation of hAMSCs. hAMSCs were cultivated for 4 weeks with rHuBMP-2 at the concentration of 200 ng/mL in a high-density monolayer culture. They demonstrated that the cells underwent morphological changes acquiring both an elongated spindle shape and a square shape. The squared cells were highly positive for “real time-polymerase chain reaction” (RT-PCR) and immunohistochemistry for aggrecan and collagen II, suggesting that chondrogenic commitment occurred. In addition, they tested the potential of hAMSCs to differentiate in vivo. They seeded hAMSCs embedded in a collagen gel supplemented with 50 ng/mL of rHuBMP-2 within a diffusion chamber that was transplanted into the abdominal muscle of mice. After five weeks, histological analysis showed the presence of round cells located in lacunae containing collagen II inside the cytoplasm and the pericellular matrix, demonstrating the capability of hAMSCs to differentiate into chondrocytes even in a non-cartilaginous environment when the right stimuli are provided (Figure 4B,D) [116].

Topoluk et al., in a recent study, compared the chondrogenic potential of hAECs, hAMSCs, and human adipose-derived stem cells (hASCs) cultured under the same conditions in vitro. The induction of differentiation was carried out by generating spheroid cultures treated with a specific chondro-inductive medium. Data showed that both hAECs and hAMSCs differentiated faster than hASCs. Indeed, hAECs and hAMSCs expressed the transcription factor *SOX9* earlier and at a higher rate and, consequently, the expression peak of *aggrecan* was also brought forward. These results were confirmed even when cells were cultivated in basal medium, suggesting that hAECs and hAMSCs possess an innate expression of chondrogenic genes. Moreover, it was evidenced that hAMSCs are more prone to commit towards the chondrogenic lineage than hAECs. As a matter of fact, hAMSCs provided the greatest amount of aggrecan, GAGs, and collagen II during ECM production (Figure 4C) [114]. Of great interest is a study from Mauro et al. that evaluated the effects of steroids in oAEC mesenchymal differentiation. The cells were treated with 12.5 µM or 25 µM Progesterone (P_4_), Oestradiol (E_2_), or with a combination of the two hormones until reaching 70–80% confluency in a monolayer. Once the confluency was reached, steroids were withdrawn, and cells were cultured in a chondrogenic differentiation medium for 21 days. The results showed that 25 µM P_4_ was able to upregulate *SOX9* but not the late chondrogenic genes, whilst 25 µM E_2_ treatment hugely enhanced the expression of chondro-related genes. Nonetheless, it should be highlighted that E_2_ effects are strictly dose-dependent and that they are negatively balanced by the simultaneous presence of P_4_ in culture. The positive effects of E_2_ on chondrogenic differentiation are related to its activity in the epithelial-to-mesenchymal transition (EMT) process, a transdifferentiation process where epithelial cells acquire a mesenchymal phenotype. Indeed, E_2_ accelerates the naturally occurring phenomenon in oAECs when cultured in vitro. EMT is activated in vitro by TGF-β-mediated signalling, which stimulates α-SMA production and the expression of *vimentin*, *twist*, and *snail* genes, typical markers of EMT. The activation of this process resulted in upregulation of *SOX9*, *aggrecan*, and *collagen IIa1* expression followed by a massive deposition of proteoglycan in the ECM, proving that E_2_ pre-treatment of AECs may be a useful tool for preparing the cells for chondrogenic stimulation (Figure 4A) [115].

In recent studies, Wang et al. evaluated for the first time, in vivo, the efficiency of a combined injection of hyaluronic acid (HA) and hAMSCs to treat osteoarthritis of the knee in a rat model. Firstly, they assessed the effects of HA on in vitro differentiation of hAMSCs. The results demonstrated that hAMSCs, treated with a combination of chondrogenic differentiation medium (containing dexamethasone, ascorbic acid, and TGF-β3) and HA (0.05 mg/mL), started the differentiation already after 7 days from the beginning of the induction, as revealed by the presence of cells showing a chondrocyte-like morphology, GAG, and collagen II deposition. Then, osteoarthritis was induced in the knee joint of a rat model. Rats were injected with HA (0.05 mg/mL) alone, hAMSCs alone, or with a combination of the two. The assessment of the joints’ regeneration state after 56 days post-injection revealed that the combination treatment allowed the production of a novel cartilage more similar to the healthy tissue in terms of morphology, matrix composition, and structure. Wang et al. demonstrated that HA and hAMSCs possess a synergistic effect, mutually influencing each other’s functions. Indeed, the presence of exogenous HA may reduce the degradation of endogenous HA and help the tissue to maintain the right healthy equilibrium of HA concentration in the ECM. Moreover, the hAMSCs, when coupled with HA, widely colonized the damaged tissue showing a high rate of survival and insertion into the joint structure. Also, a huge amount of collagen II was detected in the novel differentiated tissue, confirming that hAMSCs differentiated into functional chondrocytes. In addition, the infusion of HA and hAMSCs reduced the level of inflammation by releasing anti-inflammatory cytokines. So, Wang et al. demonstrated that an intra-articular injection of a cocktail of HA and hAMSCs may be a valuable, easy, cost-effective, and time-effective option to regenerate cartilage in an osteoarthritic rat model (Figure 4B,E) [117,118]. Also, Luo et al. furtherly demonstrated HA potential to stimulate hAMSCs chondrogenic differentiation, specifically when combined with TGF-β3 stimulus. Indeed, TGF-β3 and HA acted synergistically to induce the expression of *aggrecan* and *collagen IIa1* (Figure 4B) [72].

## 6. New Strategies to Induce Chondrogenesis

Currently, the main strategies to induce MSCs’ differentiation into chondrocytes are the use of high-density 3D cultures (i.e., spheroids) and the use of scaffolds based on several biomaterials as poly-lactic-co-glycolic acid (PLGA), polypropylene glycol (PG), hyaluronic acid, and alginate.

### 6.1. 3D High-Density Cultures

The most common culture system to induce in vitro chondrogenic commitment is the use of spheroid cultures. Spheroids are simple, unstructured, and spherical cell clusters formed via cell–cell adhesion. However, due to their simplicity, spheroids may not fully mimic the complex organization found in living tissues [119]. This strategy is particularly convenient since it resembles the condensation event, typical of the early phases of mesenchymal precursor cell commitment during embryogenesis. Indeed, cartilage development is triggered by cell-to-cell contact in the pre-cartilage condensation phase. Furthermore, it was demonstrated that N-cadherin expression is upregulated during the first phases of MSCs chondrogenic commitment in vitro. It seems that the success of differentiation depends on the aggregate density during the condensation phase. Therefore, the use of spheroids, by ensuring the maintenance of a high-density structure and reducing cell interaction with the plate plastic, is the foremost culture strategy for chondrogenic induction [59,70,113]. In fact, Winter et al. reported that cells enhanced their chondrogenic commitment when the culture was shifted from monolayer to spheroid. Moreover, they demonstrated that MSCs, after receiving the chondrogenic stimuli in monolayer, start to condensate forming high-density 3D aggregates. These aggregates express SOX9 and, consequently, start to deposit proteoglycans and collagen II on the ECM [120]. Usually, spheroid cultures are formed of about 1 *×* 10^4^–5 *×* 10^4^ cells/spheroid and put in plates containing an induction medium made up of the components described above for 21 days [110,112,114]. Miki et al. demonstrated that hAECs cultured at high density for 2 weeks increased their expression of oct-4 and nanog and formed spheroid cultures on the basal layer, suggesting that AECs in spheroid cultures retain the pluripotent markers that are reduced when cultured in monolayers adherent to the plastic surface. Then, the potential of hAECs in vitro chondrogenic differentiation was explored by evaluating the capability to differentiate towards mesodermal tissues, with highly positive outcomes [94,96,97]. Also, greater efficiency of 3D spheroid cultures in stimulating chondrogenesis was proved in hAMSCs. For instance, Teng et al., using immortalized chondrogenically-induced hAMSCs, demonstrated that high-density cultures promoted the expression of SOX9, collagen II production, and generation of a membranous structure that resembled the perichondrium [121]. Muiños-López et al. further proved the efficiency of this culture strategy for chondrogenic differentiation of both hAECs and hAMSCs. They covered high-density cultures of hAECs and hAMSCs with hAM and demonstrated that differentiated hAMSCs synthesized the highest amounts of ECM and collagen II, even when compared to chondrocytes and hBM-MSCs [122].

### 6.2. Role of 3D Scaffold in Stem Cell Chondrogenic Differentiation

Biomaterial scaffolds play a crucial role in directing stem cell differentiation by mimicking the role of their niche’s ECM. In chondrogenic induction, biomaterial engineering strives to emulate the physical and biochemical properties of cartilage niche using materials such as alginate, hyaluronic acid, collagen, agarose, PG, and PLGA [108,123,124]. Natural polymers, preferred for cartilage engineering, create a hydrated 3D networks and possess low immunogenicity [125]. Research frequently concentrates on integrating multiple biomaterials in scaffold fabrication. For instance, in several studies, collagen scaffolds were linked with GAGs, cellulose, or alginate to increase the reliability of the scaffold [70,126]. In a study by Wei et al., the ability of hAMSCs to differentiate in vivo was assessed by implanting hAMSCs seeded on a collagen I scaffold and treated with BMP-2 into nude rat models of cartilage defects. The construct was engrafted into a cartilage lesion artificially produced on the medial femoral condyle. Two months after transplantation the lesioned zone showed the presence of white to pink soft tissue covering the entire lesion. Histological analysis demonstrated that the tissue contained chondrocytes that were also confirmed to be alive and that collagen II was released into the ECM [116]. In a study by Saghati et al., a phenolated alginate (Alg Ph)/collagen hydrogel was proposed as an innovative biomaterial for AMSCs chondrogenic induction. Alginate hydrogels are inert biomaterials since receptors for alginate do not exist on the cell surface. Consequently, alginate biomaterials have to be necessarily linked to an adhesion molecule as collagen. In this study, further modification with phenolic hydroxyl moieties led to greater biocompatibility and cellular attachment. They reported that the proposed hydrogel possesses a highly homogeneous porosity and mechanical stability (i.e., high compression strength) thanks to the presence of collagen and phenol, respectively. The greatest property of hydrogels is their ability to exchange water and regulate their volume; in this way, hydrogels ensure the right apport of nutrients and growth factors to the cells cultured within the scaffold. The Alg-Ph/Col hydrogel possesses an appropriate level of permeability thanks to the presence of collagen, which reduces the mean pore size. Moreover, hAMSCs treated for 21 days in this hydrogel demonstrated an elevated survival rate and synthesized SOX9 and collagen IIa, even in the absence of an induction medium [111].

A valuable alternative source to produce scaffolds is the AM, a naturally occurring matrix that can be easily manipulated as a scaffold for tissue engineering and as a cell carrier thanks to its anti-fibrotic, antibacterial, anti-inflammatory, biocompatible, low immunogenic, and significant mechanical properties [106,127]. It appears to be the most suitable scaffold for cartilage tissue engineering, since its ECM naturally occurs with essential components of cartilage ECM such as collagen (I, III, IV, V, and VI), hyaluronan, and proteoglycans, fundamental for maintaining chondrocytes’ phenotype and metabolism. The AM contains numerous growth factors, such as TGF-β, “Platelet-derived growth factor” (PDGF), and basic FGF (bFGF), that, along with physical cues, provide a pro-regenerative environment, stimulating rapid tissue regeneration/repair and cell proliferation. Another feature making AM a good scaffold is its natural biocompatibility thanks to its immunomodulatory properties, which reduce the risk of transplant rejection and of graft vs. host disease and the rapid self-biodegradation occurring in 3–4 weeks [106,128,129,130]. Thanks to the natural production of antibacterial molecules, AM protects against bacterial invasion. Its capability to firmly attach to surfaces eases the surgeons in the application of grafts on tissue surfaces without the need for suturing [128,129]. Jin et al. have been the first to test the feasibility of using the AM as a scaffold for chondrocytes in the treatment of cartilage lesions, both in vitro and in vivo. They used a decellularized matrix seeded with chondrocytes and cultured for 4 weeks on the three different layers of AM: the epithelial layer, the stromal layer, and the basement membrane layer. They evaluated at two timepoints (1 week and 4 weeks) the proliferation rate and the phenotype features. It was demonstrated that only the cells seeded on the stromal layer were able to migrate the entire depth of the scaffold, maintaining the typical round shape. Moreover, the stromal group was the one that accumulated the highest levels of collagen II over time, where the protein synthesis was stronger than in the other groups. Consistent with these in vitro results, Jin et al. chose the stromal layer for in vivo experiments on a rabbit model for osteochondral defects. They demonstrated that hyaline cartilaginous tissue was obtained in rabbits at 4 weeks from surgery and that the damaged area was completely repaired with mature hyaline cartilage at 8 weeks, concluding that the AM represents a crucial naturally derived biomaterial for cartilage tissue engineering [106]. Later, Tan et al. proposed the use of AM scaffolds to support and improve the chondrogenic commitment of MSCs. They studied the efficacy of processed AMs (air-dried or freeze-dried) for inducing MSCs chondrogenic differentiation. The results showed that MSCs successfully attached and proliferated on the membrane, probably thanks to the huge amount of hyaluronan naturally occurring in the AM, which promotes cell–matrix interaction (CD44-hyaluronan binding) and stimulates chondrogenesis. Furthermore, the high porosity of the freeze-dried AM stromal layer allowed the MSCs to migrate throughout the whole structure. Indeed, MSCs seeded on the freeze-dried AM possessed a phenotype more similar to chondrocytes than on air-dried AM and a higher amount of GAG production was detected, concluding that AM may be a good scaffold material for chondrogenic differentiation of MSCs [130]. Lindenmair et al. proposed a further innovation in the use of hAM as a scaffold, that is, the in vitro differentiation of hAECs and hAMSCs into chondrocytes using hAM as a supporting material. hAECs and hAMSCs were stimulated using a chondrogenic differentiation medium with or without FGF-2 or with a chondrocyte redifferentiation medium for 56 days. The researchers showed that the groups treated with chondrogenic differentiation medium plus FGF-2 were highly positive to Alcian Blue staining and that GAGs and collagen II were accumulated. The cells treated were mostly found in the mesenchymal layer where the matrix appeared denser and more homogenous, the cells took a spherical shape and resided inside lacunae-like structures. They also proved that SOX9 expression was low in all the tested conditions, whilst COMP, an early marker of chondrogenesis, was strongly upregulated in chondrogenic-media-treated groups. Unfortunately, “chondroitin sulfate proteoglycan” (CSPG) 2, a marker of fibrocartilage, and “calcium-dependent mitochondrial aspartate and glutamate carrier” (AGC)1, a marker of hyaline cartilage, were also upregulated and downregulated, respectively, indicating that chondrogenic differentiation of hAM-derived cells in these conditions may not lead to hyaline cartilage but to the production of a fibrocartilaginous tissue. Moreover, the epithelial layer was not conserved in these culture conditions. In contrast, the redifferentiation medium seemed useful for the maintenance of AM-derived cell viability, mostly supporting hAECs, which are the most prominent cellular type in hAM. These results suggest that hAMSCs may be more suitable for chondrogenic differentiation strategies, being probably the first line of differentiation when cultured on the AM. Lindenmair et al. concluded that hAM cultivated with its cells can be a valuable option for cartilage tissue engineering, easing and reducing the time and costs of the procedure [131]. More recently, Naseer et al. studied the feasibility of differentiating placenta-derived stem cells and umbilical-cord-derived stem cells into chondrocytes on denuded hAM. Stem cells were cultured on the basement side of the hAM and the efficiency of hAM as a scaffold for chondrogenic differentiation was compared with that of a plastic adherence surface. On both scaffolding materials, placenta-derived stem cells and umbilical-cord-derived stem cells changed their morphology (binucleated polygonal and hexagonal cells) and formed cell aggregates after chondrogenic induction. It was found that morphological changes were parallelly followed by an increase in the proteoglycan, aggrecan, and collagen content of cells. Naseer and colleagues chose the basement side instead of the stromal side due to its great leading on cell attaching and proliferative capability. The presence of typical cartilage ECM biomolecules such as collagen and proteoglycans in the hAM matrix made it a better supportive matrix for stem cell differentiation compared to the plastic adherence surface, suggesting that hAM represents an efficient and useful support for placenta-derived chondrogenic differentiation, making it an appealing scaffold material for grafts for articular cartilage diseases [127]. In an interesting in vivo study, Rastegar Adib et al. evaluated the use of decellularized ECM (dECM) from ovine osteochondral tissue coupled with platelet-rich fibrin, AM extracts, or BM-MSCs to treat osteochondral defects in rabbits. They chose the ECM from osteochondral tissue as a scaffold for its dual cartilaginous/bone biomechanical properties and its native tissue polymer arrangement. Furthermore, the coupling of this natural scaffold with biological materials enhances and fastens the healing reparation capacity, furnishing better regeneration outcomes. Data from this study demonstrated that coupling with AM extracts provided the greatest results for osteochondral regeneration. Indeed, the higher positivity to Safranin-O staining and macroscopic observation of the graft 3 months post-surgery proved the formation of functional hyaline cartilage in the group treated with dECM plus AM extracts [132]. You et al. proposed the production of hAMSCs sheets as scaffolds for repairing osteochondral defects. They prepared hAMSCs sheets that were cultured in a chondrogenic differentiation medium and encapsulated cartilage particles. After induction, hAMSCs produced huge levels of aggrecan and collagen II in the ECM, and the cells acquired a round morphology and were closely arranged and homogeneously distributed. The hAMSCs sheets supplemented with cartilage particles were implanted in rabbits. After 3 months from surgery, the reparative tissue completely replaced the lesion, producing a tissue that resembled, both in colour and structure, the healthy articular cartilage; aggrecan and collagen II were also synthesized. This regenerated tissue successfully integrated with the native cartilage and the subchondral bone. So, the hAMSCs sheets coupled with cartilage particles successfully regenerated cartilage and repaired osteochondral defects in rabbit animal models. The success of this strategy is probably attributed to the paracrine effects of hAMSCs, which may have attracted progenitor cells from the surrounding environment to participate in the regeneration process. Also, hAMSCs may have released growth factors, such as TGF-β, that further promoted the migration and proliferation of chondrocytes into the lesion. The implantation of a cell sheet may even offer protection from the cytokines produced by the damaged cartilage, preventing the loss of cartilaginous ECM [107].

### 6.3. New Perspectives: Organoids

In recent years, research on novel 3D in vitro models has gained the attention of the scientific community. The purpose is to produce models that are more advanced and better resemble human tissues compared to in vivo models [133,134,135]. In this field, organoids emerged as a promising approach. Organoids are complex, organized, microscopic 3D tissue models made up of stem cells, usually MSCs or induced pluripotent stem cells (iPSCs) that attempt to replicate the structure and function of a specific organ [135,136,137,138]. They comprise multicellular components that produce an ECM, resembling the stem cell niche. Importantly, organoids, if cultured under precise culture conditions, differentiate toward the desired cytotypes, highly mimicking the tissue to be studied. Furthermore, one of organoids’ main advantages is the possibility to use human and patient-specific cells, allowing the application of personalized medicine. Moreover, they reduce the cost and the research time compared to the classical animal models. Until now, organoid cultures have been used for the study of many human tissues and organs, such as the pancreas, lungs, and liver, but few efforts have been made in the study of the skeletal system [133,134,135]. Unfortunately, to our knowledge, no attempt has been made to generate organoids resembling the skeletal system with AECs and AMSCs. Consequently, new lines of research should be carried out to apply this innovative culture system to the study of AECs’ and AMSCs’ chondrogenic differentiation, since it may pave the way to new solutions for musculoskeletal regenerative medicine in the clinical field.

## 7. Conclusions

During the past years, research has focused on validating in vitro and in vivo strategies to generate cartilage tissue and to improve mechanisms controlling chondro-healing. Currently, few cell-based techniques have been approved in the clinics, such as ACI and Spherox. Unfortunately, these techniques are not free from drawbacks. For instance, ACI requires two surgical operations and a long post-operation recovery. Yet, the main shortcoming of ACI is the unavoidable chondrocyte de-differentiation during their in vitro 2D expansion. This shortcoming was overcome using the 3D strategies such as Spherox, yet Spherox still needs the surgical operation to be performed and it is applicable just for the treatment of wide lesions. Several other techniques have been proposed during the years, but none of them succeeded in completely satisfying the huge requirements for novel functional cartilaginous tissue generation. To date, the FDA and EMA have approved only the use of chondrocytes as a cell source for the clinics. At any rate, other stem/progenitor cell sources deemed more suitable for tissue engineering techniques are under evaluation [17,18,19,139], AECs and AMSCs have particularly caught the scientists’ attention [26,94,95]. Innovative stem cell sources can, indeed, surmount the main drawbacks of the current techniques approved for cartilage repair. Indeed, they allow a reduction in the number of chondrocytes required, thus limiting the donor site morbidity concern. Moreover, they can promote the targeted repair of cartilage lesions by creating a regenerative environment through the release of pro-regenerative factors and by recalling endogenous stem cells from surrounding tissues, mimicking the in vivo niche [140,141]. To understand how those cells differentiate towards the chondrogenic lineage and, consequently, develop efficient differentiation strategies, it has been necessary to focus on the molecular pathways involved in chondrogenesis. It is clear that chondrogenesis is a complex process, resulting from the simultaneous cooperation and equilibrium among different agonist and antagonist molecular pathways [67,69]. Numerous in vitro strategies have been proposed to differentiate AECs and AMSCs into the chondrogenic lineage; among those, 3D strategies are the most convenient for chondrogenic differentiation of stem cells, since they better reproduce the event of condensation during early embryogenesis [67,70,111]. Moreover, the use of scaffolds that reproduce the cartilaginous matrix as hydrogel or the AM itself further stimulates the process [111,127]. To conclude, several steps have been taken to understand the complex process of chondrogenesis and to reproduce it in AMSCs and AECs, in order to develop new treatments for such a burdening disease as osteoarthritis. Yet, great effort is still needed in the near future to find the right formula for satisfying in vitro and in vivo use of AECs and AMSCs. Nevertheless, these cells represent an innovative strategy for the production of functional mature cartilaginous tissue.

## Figures and Tables

**Table 1:** In vivo KO models to study chondro-inductive pathways.

KO Models	Target	Effects	References
mEmbryo chimeras from *SOX9-/-* embryonic stem cells	*SOX9*	Lack of chondrogenic condensation Lack of late chondrogenesis markers (aggrecan and COL2a1)	Bi et al., 1999 [30]
*mSOX5-/-* and *SOX6-/-*	*SOX5* and *SOX6* (downstream to SOX9)	Chondrodysplasia No chondrocytes differentiation: lack of cartilage	Smits et al., 2001 [31]
mConditionally *SOX9-/-*	*SOX* trio genes	Before condensation: Lack of chondrogenic condensation Lack of cartilage Absence of *RUNX*2 expression: no bone development Absence of *SOX*5 and *SOX*6 expression After condensation: Chondrodysplasia ↓ *Noggin*: lack of *Gdf5* and *Wnt14* expression Hypertrophy ↓ Ihh pathway: lack of endochondral ossification Absence of *SOX5* and *SOX6* expression	Akiyama et al., 2002 [32]
m*SMAD3(ex8/ex8)* KO	TGF-β/SMAD3 signalling pathway	Alterations during late phases of chondrogenesis: Cartilage degeneration: osteoarthritis ↑ chondrocytes differentiation: hypertrophy Osteophytes ↓ Proteoglycans	Yang et al., 2001 [33]
m*SMAD3-/-*	TGF-β/SMAD3 signalling pathway	↑ Chondrocytes differentiation: - ↑ *Col X* - ↑ BMP pathway Hypertrophy: prevented by administrating TGF-β and Noggin ↑ endochondral ossification after fracture: ↑ apoptotic cells + osteoclasts ↓ *SOX9* expression in the fractured site	Li et al., 2005 [34] Kawakatsu et al., 2011 [35]
mMT-DNIIR (KO for *TGFRII*)	TGF-β signalling pathway	Hypertrophy ↑ *ColX* ↑ Ihh pathway ↓ Proteoglycans Osteoarthritis: - Presence of osteophytes - Fibrillation of cartilage - ↑ Proliferation of chondrocytes and clustering	Serra et al., 1997 [36]
m*BMP2-/-* and *BMP4-/-* embryos	BMP signalling pathway	Lethal during embryogenesis	Winnier et al., 1995 [37] Zhang et Bradley. 1996 [38]
mConditionally *BMP2-/-* and *BMP4-/-* or *BMP2-/-* and *BMP7-/-*	BMP signalling pathway	Lack of osteoblastogenesis ↓ Skeletal elements size in limbs Delayed chondrogenesis *BMP2/BMP4* deletion is the most crucial	Bandyopadhyay et al., 2006 [39]
m*SMAD1-/-* and *SMAD5-/-*	BMP/SMAD1/SMAD5 canonical signalling pathway	Impairment of chondrogenesis: blocking of chondrocytes differentiation Severe chondrodysplasia Impairment of Ihh pathway: - Premature hypertrophy - Growth plate disorganization - Lack of osteoblasts	Retting et al., 2009 [40]
m*BMPR1B-/-* and conditionally *BMPR1A-/-*	BMP signalling pathway	KO for *BMPR1B*: Compatible with life Defects in distal phalanges KO for *BMPR1A*: Skeletal dysplasia Cartilage hypertrophy Double KO (*BMPR1B* and *BMPR1A)*: Severe generalized chondrodysplasia Lack of chondrogenic precursors differentiation Lack of chondrocytes proliferation, survival and, differentiation Delayed ossification: ↓ *COL X* Accumulation of hypertrophic chondrocytes	Yoon et al., 2005 [41] Yoon et al., 2006 [42]
m*Noggin-/-*	Noggin-mediated BMP pathway antagonism	Absence of joints Excessive amounts of cartilage ↑ BMP activity Oversized growth plates: - Excessive recruitment of progenitor cells into cartilage - Absence of joint sites specification Hyperplasia No effects during early phases of osteochondrogenesis	Brunet et al., 1998 [43]
m-*β-catenin-/-*, *LRP5-/-*, and *LRP6-/-* mice	Wnt/β-catenin signalling pathway (canonical pathway)	Defective cartilage anlagen Shortening of endochondral bone Lack of hypertrophic chondrocytes ↓ *Col2a1*	Joeng et al., 2011 [44]
m-*β-catenin-/-*	Wnt/β-catenin signalling pathway (canonical pathway)	Loss of chondroprogenitor cells in the growth plate Deformed and disorganized growth plate	Candela et al., 2014 [45]
m*Wnt5a-/-*	Non-canonical Wnt signalling pathway	Delayed transition from proliferative to hypertrophic stage of chondrocytes: ↑ *SOX9* expression Short and thick cartilage Loss of columnar organization of chondrocytes	Yang et al., 2003 [46]
m*FGFR3-/-*	FGF signalling pathway	Impaired hypertrophic process	Colvin et al., 1996 [47] Deng et al., 1996 [48]
zebrafish *FGFR3-/-*	FGF signalling pathway	Craniofacial malformation: - Microcephaly - Dysregulation of cartilage development (↑ hypertrophy, chondrodysplasia, impaired chondrocytes’ arrangement) - ↓ Ihh pathway - ↓ Wnt/β-catenin pathway	Sun et al., 2020 [49]
m*HIF1A-/-*	Hypoxia response pathway	Lethal mutation → mice die during gestation	Iyer et al., 1998 [50]
mConditionally *HIF1A-/-*	Hypoxia response pathway	Death of chondrocytes in the centre of cartilage ↑ p57: delayed hypertrophic differentiation Osteoarthritis ↑ Wnt/β-catenin pathway Activation of MMP13 ↑ Pro-catabolic enzymes ↑ Number of hypertrophic chondrocytes ↓ Limb length	Schipani et al., 2001 [51] Bouaziz et al., 2016 [52]
m*HDAC4-/-*	Epigenetic modifications	Faster ossification Ectopic bone production ↑ Number of hypertrophic chondrocytes ↑ Ihh pathway ↑ *RUNX2*	Vega et al., 2004 [53]
m miR-455-3p-/-	Epigenetic modifications	Osteoarthritis Irregular and hypocellular joints ↑ MMP13 ↑ PTEN: inhibition of PI3K/AKT Inhibition of TGF-β/SMAD pathway ↓ *Col2a1*	Hu et al., 2019 [54] Wen et al., 2020 [55]
m miR-140-/-	Epigenetic modifications	Osteoarthritis Degeneration of joints Loss of proteoglycans Cartilage fibrosis Short height Cranial malformation ↑ Matrix degrading enzymes ↓ Proliferative chondrocytes ↓ BMP activity ↓ Chondro-related genes	Miyaki et al., 2010 [56] Nakamura et al., 2011 [57]

Knock out (KO); mouse (m); SRY-Box Transcription Factor (SOX); Collagen (COL); Runt-related Transcription Factor 2 (RUNX2); Growth Differentiation Factor 5 (GDF5); Wingless-related integration site (Wnt); Small Mothers Against Decapentaplegic (SMAD); exon (ex); Transforming Growth Factor-beta (TGF-β); Bone Morphogenetic Protein (BMP); Dominant-Negative Type II TGF-beta Receptor (MT-DNIIR); Indian Hedgehog (Ihh); Bone Morphogenetic Protein Receptor Type 1A (BMPR1A); Bone Morphogenetic Protein Receptor Type 1B (BMPR1B); Low-Density Lipoprotein Receptor-related Protein (Lrp); Fibroblast Growth Factor Receptor 3 (Fgfr3); Hypoxia-Inducible Factor 1 Alpha (HIF1α); Matrix Metalloproteinase (MMP); Histone Deacetylase 4 (HDAC4); MicroRNA (miR); Phosphatase and Tensin Homolog (PTEN); Phosphoinositide 3-Kinase (PI3K); Protein Kinase B (AKT); ↓: Decrease; ↑: Increase.

## Data Availability

Not applicable.
